# Editorial: Voice analysis in healthy subjects and patients with neurologic disorders

**DOI:** 10.3389/fneur.2023.1288370

**Published:** 2023-09-28

**Authors:** Antonio Suppa, Giovanni Costantini, Pedro Gomez-Vilda, Giovanni Saggio

**Affiliations:** ^1^Department of Human Neurosciences, Sapienza University of Rome, Rome, Italy; ^2^IRCCS Neuromed Institute, Pozzilli, Italy; ^3^Department of Electronic Engineering, University of Rome Tor Vergata, Rome, Italy; ^4^Center for Biomedical Technology, Universidad Politécnica de Madrid, Madrid, Spain

**Keywords:** voice analysis, machine-learning, Parkinson's disease, mild cognitive impairment, stuttering, multiple sclerosis

Voice is a complex biological phenomenon resulting from the highly integrated and coordinated activation of the phonatory, respiratory and articulatory apparatus achieved by the nervous system. Voice is currently receiving growing interest among researchers working in the field of neurology. The neuronal basis of voice is indeed a fascinating area of research that explores the intricate neural networks involved in the production of vocalizations and is currently termed the phonological loop. Besides the complexity of brain networks contributing to vocalization, the acoustic analysis of voice is also characterized by high-dimensional data based on an exponential number of features. Accordingly, conventional voice analysis has recently received a relevant boost from more advanced approaches based on artificial intelligence and machine learning algorithms. By rapidly processing vast amounts of data, artificial intelligence can identify subtle patterns and anomalies that might be overlooked by human observers, leading to faster and more precise classifications. Indeed, recent studies have demonstrated that machine-learning algorithms significantly improve the accuracy of the objective classification of voice samples in humans [(1–4); Asci et al.]. By providing non-invasive and cost-effective tools, voice analysis based on artificial intelligence holds significant potential to revolutionize the clinical management of neurologic disorders manifesting in voice abnormalities, as well as in a telemedicine scenario.

The present Research Topic entitled *Voice Analysis in Healthy Subjects and Patients with Neurologic Disorders* is an updated collection of research in the field. The first set of studies included in the Research Topic has investigated voice abnormalities in people with Parkinson's disease (PD) and other movement disorders. Two studies have confirmed and further expanded previous findings supporting the robustness and native language-independent accuracy of conventional as well as automated voice analysis based on artificial intelligence in people with PD (dos Santos et al.; Favaro et al.; Scimeca et al.). The observations strongly support the reliable use of voice analysis in people with different native languages (5). Moreover, concerning the interpretation of voice changes in people with PD, Cavallieri et al. have demonstrated a significant correlation between voice abnormalities and the severity of upper bradykinesia in advanced PD thus raising new pathophysiological hypotheses. A second set of studies has examined the link between cognitive functions and specific voice features. Taptiklis et al. have clarified the modulatory effect of mental effort on voice, whereas Higuchi et al. have reported voice abnormalities related to mild cognitive impairment. These findings overall point to the complexity of the modulatory effect of higher cortical functions in healthy subjects and people with physiological aging or mild cognitive impairment (1). A final set of studies has demonstrated voice changes in people with multiple sclerosis (Kieling et al.) and stuttering (Asci et al.), supporting the utility of objective voice analysis in the overall clinical management of various neurologic conditions.

In conclusion, the research articles included in the present Research Topic further support the clinical utility of objective voice analysis in people with Parkinson's disease and other neurologic disorders. Voice analysis presents an evolving and promising non-invasive and cost-effective tool to complement traditional assessments, potentially leading to earlier diagnoses and improved outcomes for patients with various neurologic conditions (6, 7). However, the future implementation of voice analysis in a clinical scenario should prioritize patient privacy and ethical considerations to ensure the responsible and secure use of voice data for healthcare purposes.

## Author contributions

AS: Writing—review and editing. GC: Writing—review and editing. PG-V: Writing—review and editing. GS: Writing—review and editing.
